# Biopolymer-Based Nanoparticles for Cystic Fibrosis Lung Gene Therapy Studies

**DOI:** 10.3390/ma11010122

**Published:** 2018-01-13

**Authors:** Elena Fernández Fernández, Beatriz Santos-Carballal, Chiara de Santi, Joanne M. Ramsey, Ronan MacLoughlin, Sally-Ann Cryan, Catherine M. Greene

**Affiliations:** 1 Lung Biology Group, Department of Clinical Microbiology, Royal College of Surgeons in Ireland, Beaumont Hospital, Dublin 9, Ireland; chiaradesanti@rcsi.ie (C.d.S.); cmgreene@rcsi.ie (C.M.G.); 2 ChiPro GmbH, Anne-Conway-Straße 1, 28359 Bremen, Germany; bcarballal@chipro.de; 3 School of Pharmacy, Royal College of Surgeons in Ireland, Dublin 2, Ireland; joanneramsey@rcsi.ie (J.M.R.); RMacLoughlin@aerogen.com (R.M.); scryan@rcsi.ie (S.-A.C.); 4 School of Pharmacy and Pharmaceutical Sciences, Trinity College, Dublin 2, Ireland; 5 Aerogen Ltd., Galway Business Park, Dangan, Galway H91 HE94, Ireland

**Keywords:** cystic fibrosis, cystic fibrosis transmembrane conductance regulator (CFTR), lung gene delivery, nanoparticles, biopolymers, PLGA, chitosan, Locked-Nucleic Acid (LNA)

## Abstract

Lung gene therapy for cystic fibrosis disease has not been successful due to several challenges such as the absence of an appropriate vector. Therefore, optimal delivery of emerging therapeutics to airway epithelial cells demands suitable non-viral systems. In this work, we describe the formulation and the physicochemical investigation of biocompatible and biodegradable polymeric nanoparticles (NPs), including PLGA and chitosan (animal and non-animal), as novel methods for the safe and efficient delivery of CFTR-specific locked nucleic acids (LNAs).

## 1. Introduction

The most frequent lethal genetic disease in Caucasian populations is Cystic Fibrosis (CF). Mutations in the *cystic fibrosis transmembrane conductance regulator* (CFTR) gene cause abnormal ion transport in the epithelium of several tissues, which results in the production of abnormally thick and sticky mucus that blocks the organ, principally the lung, and is responsible for CF pathology. Chronic inflammation and recurrent bacterial infections are the result [1], leading to the progressive destruction of lung tissue and making pulmonary disease the primary cause of mortality in CF [2].

Correction of the defective CFTR gene is an attractive solution for this single-gene disease. Successful gene transfer formulations depend on two components which are: the therapeutic nucleic acid and a carrier molecule that binds to or contains that nucleic acid. However, clinical approaches for CF genetic therapies have mostly failed due to increased immune responses towards the vectors. Gene therapy focused on the use of viral carriers has been widely studied in CF treatments due to the high transfection efficiency reported [3]. Nevertheless, the use of viruses as vectors raises many concerns regarding possible immune responses, biosafety and severe inflammation after long periods of administration [4]. Therefore, non-viral vectors have emerged as a potentially safer alternative [5].

The use of biocompatible and biodegradable polymers such as chitosan or polylactide-co-glycolic acid (PLGA) is becoming more common for the next generation of nanoparticles. Cationic polymers bind to negatively charged nucleic acids through electrostatic interactions to form polyplexes. Chitosan is the main derivative of chitin, the second most abundant polysaccharide in nature. It is a linear biodegradable polysaccharide composed of randomly distributed β(1-4)-linked-d-glucosamine and *N*-acetylglucosamine units. Chitosan exhibits several properties that make it an interesting material for pharmaceutical formulations. It induces low cytotoxicity, is biocompatible, biodegradable and mucoadhesive [6,7,8]. All these properties place chitosan as a potential alternative to administer therapeutics to the lung. The cationic properties of chitosan are useful in pharmaceutical formulations and biomaterials because the molecule can form polyelectrolyte complexes with negatively charged DNA, RNA, siRNA and microRNA [9]. It has been reported as a suitable candidate for transmucosal administration [10] and, after intratracheal administration to mice, chitosan complexes were found in the mid-airways and transgene expression was observed in epithelial cells [11]. Gene therapy based on the use of chitosan as a delivery vector has been extensively considered in the last decade [12], but few studies have assessed the use of chitosan as a potential gene delivery vector for CF [13,14,15,16].

Besides this natural polymer, the synthetic polymer PLGA is approved by the US Food and Drug Administration (FDA) and European Medicine Agency (EMA) in various drug delivery systems in humans [17]. PLGA polymers are widely used due to their inherent biocompatibility, low toxicity, immunogenicity, and biodegradability [17,18]. In vivo, PLGA is hydrolyzed to lactic acid and glycolic acid which are subsequently removed via the citric acid cycle. By altering the ratio of each monomer, different forms of PLGA can be produced with different rates of degradation. PLGA 50:50 (i.e., a copolymer whose composition is 50% lactide and 50% glycolide) is the most widely used copolymer, with the fastest biodegradation rate, which degrades in about 50 to 60 days [19]. PLGA drug carriers can achieve sustained cytoplasmic delivery via rapid escape from endolysosomes and can be used to generate a wide range of particles from nanoparticle (~10–1000 nm) to microparticle (1–250 µm) sizes. Consequently, PLGA nanoparticles encapsulating nucleic acids can be developed for both inhalation and targeting to the lower respiratory tract [20].

MicroRNAs (miRNA) are regulatory RNAs that negatively regulate target gene expression. CFTR is known to be regulated by a set of lead miRNAs which are overexpressed in the lungs of people with CF [21,22,23,24]. A new therapeutic strategy for CF involves cytoplasmic delivery of Locked Nucleic Acid (LNA)-modified RNA oligonucleotides custom-designed to block endogenous miRNAs binding to their cognate miRNA recognition elements (MREs) in the 3′ untranslated region (UTR) of the CFTR messenger RNA. These custom-designed inhibitors termed Target Site Blockers (also commonly referred to as morpholinos) do not have off-target effects associated with antimiR inhibitor approaches [25]. Anti-miRNA LNAs are antisense oligonucleotides that can be synthesized to be complementary to a mature miRNA of interest and inhibit its function [26]. Once introduced into cells, they act by masking the miRNA target site while not affecting the activity of the endogenous miRNA *per se*. LNAs possess an extremely high binding affinity to complementary RNA oligonucleotides, display improved mismatch discrimination and show high stability in biological systems [27].

Here we report a comprehensive investigation of polymeric nanoparticles (NPs) as a novel method for the safe and efficient delivery of CFTR-specific LNAs. To this aim, particles were prepared and characterized with state-of-the-art techniques, loading efficiency and stability in biological environment was assessed as well as their stability after nebulization.

## 2. Results

### 2.1. Physicochemical Characterization by Dynamic Light Scattering (DLS) and Nanoparticle Tracking Analysis (NTA)

NPs were prepared using PLGA loaded with specific LNA oligonucleotides at N/P ratio 4 following the protocol described by Kelly and coworkers [20]. Regarding NPs from chitosan sources, systems were prepared from a stock solution containing acetic acid and LNAs. Complexes with different N/P ratios (1.5, 2, 5, 10, 15) were formed.

**Figure 1:**
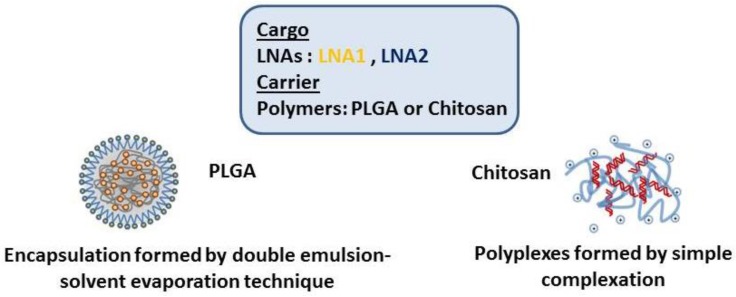
Graphic representation of the different nanoparticles prepared.

A graphic representation of both types of systems is shown in Figure 1. Table 1 describes the physicochemical characteristics of the biopolymers. The LNAs used during this study (LNA1 and LNA2) were 16 bases in length with a 38% GC content. Since optimal particle size and colloidal stability in suspension are important aspects to consider, all NPs were fully characterized in terms of their average size diameter, polydispersity and surface charge (zeta potential). 

**Figure 2:**
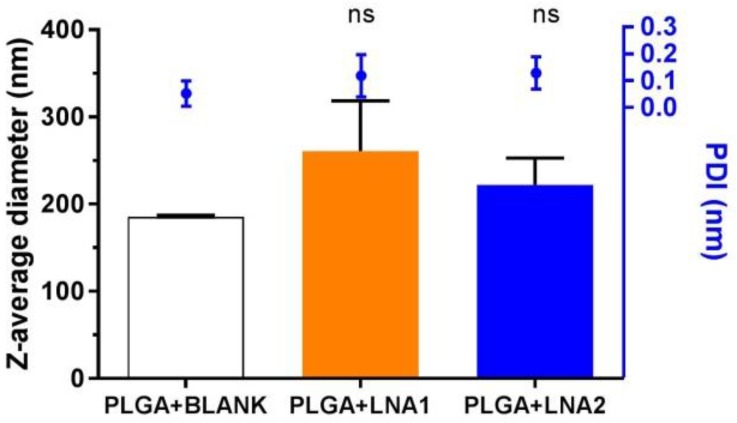
Variation of the Z-average size hydrodynamic diameter (nm) and polydispersity index (PDI) of PLGA NPs formed with LNA1 and LNA2. The values represented are the mean averages ± SD of three independent experiments. Statistical comparisons were performed between unloaded and loaded PLGA particles using Kruskal–Wallis test for non-parametrical distribution (*p* < 0.05); “ns” stands for non-significant differences.

**Figure 3:**
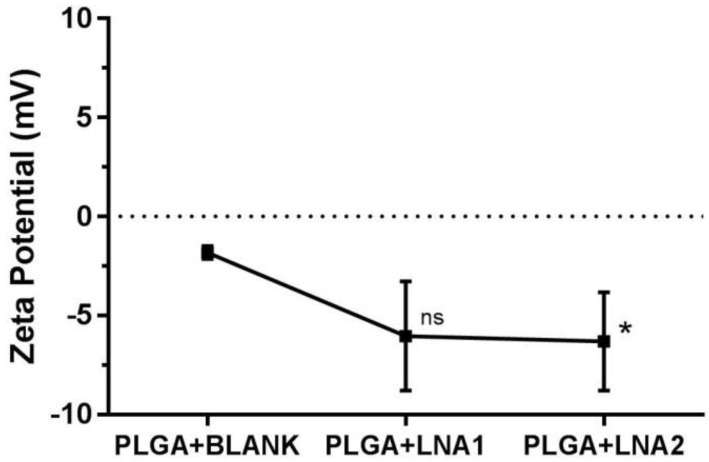
Variation of the zeta potential (mV) of PLGA NPs formed with LNA1 and LNA2. The values represented are the mean ± SD of three independent experiments. Statistical comparisons were performed between unloaded and loaded PLGA particles using Kruskal-Wallis test for non-parametrical distribution (* *p* < 0.05); “ns” stands for non-significant differences.

Figure 2 shows the values of average size diameter and polydispersity index (PDI) for the nanoparticles coated with PLGA obtained by DLS. The particle size oscillates between 180 and 300 nm, and non-significant differences in size were found after the loading of the particles with the LNA. The PDI value was of approximately 0.1 for all the systems. It is suggested that PDI values below 0.3 are desired in order to ensure monodisperse nanoparticle formulations [28]. Higher size values were seen for particles encapsulating the LNA oligonucleotides. Specifically, PLGA particles loaded with LNA1 and with LNA2 present a Z-average diameter of 260 ± 60 nm and 222 ± 30 nm, respectively. In comparison, Nanoparticle Tracking Analysis (NTA) measurements determined the size averages to be 166 ± 2 nm and 168.3 ± 0.3 nm respectively (Table 2). The measured ζ-potential, corresponds to the difference in electric potential generated between the particle surface and the medium in which they are dispersed. Figure 3 depicts the variation of the zeta potential of PLGA NPs. Unloaded (blank) particles have a slightly negative ζ-potential value. Particles loaded with LNA1 and LNA2 show a minor reduction in the ζ-potential to values of around −7 mV for both. 

**Figure 4:**
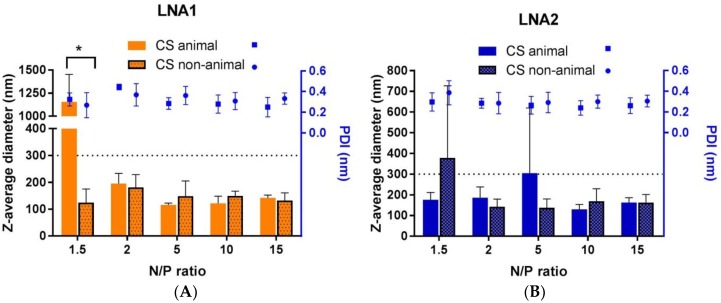
Variation of the Z-average size hydrodynamic diameter (nm) and polydispersity index (PDI) of chitosan (animal and non-animal) NPs formed with LNA1 (**A**) and LNA2 (**B**) at different N/P ratios (1.5, 2, 5, 10, 15). The values represented are the mean averages ± SD of three independent experiments. Statistical comparisons were done between particles formed with either animal CS or non-animal CS within the same ratio and LNA. Statistical significance (*) was determined using the Holm-Sidak method, with alpha = 5.0%.

**Figure 5:**
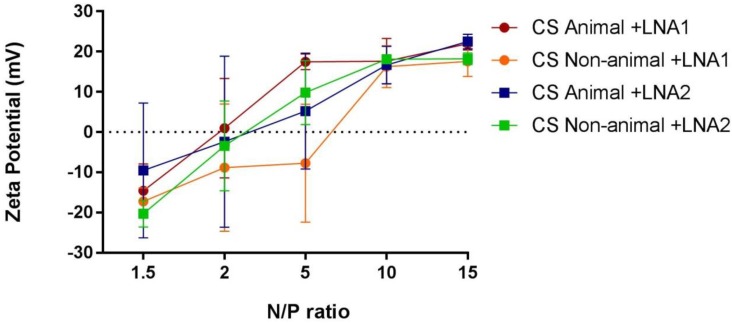
Variation of the zeta potential (mV) of chitosan (animal and non-animal) NPs formed with LNA1 and LNA2 at varying N/P ratios (1.5, 2, 5, 10, 15). The values represented are the mean ± SD of three independent experiments

Likewise, the particles formed by simple coacervation processing using chitosans were characterized by DLS. Figure 4 shows two graphics corresponding to the average size hydrodynamic diameter and polydispersity index (PDI) of complexes formed with either LNA1 or LNA2. Complexes were designed under a range of positive N/P ratio values, specifically, 1.5, 2, 5, 10, 15. DLS technique revealed that all the complexes formed with non-animal chitosan have a size below 200 nm and the small variations in size do not correlate to the different N/P ratios. Complexes formed with chitosan from animal source loading LNA1 presented agglomerates at a N/P ratio of 1.5, and this is statistically significant. At ratio of 5 when bound to LNA2 the size increased up to 300 nm. In the case of NTA measurements, all CS systems present an average size under 200 nm (see Table 2). The values of PDI in all cases are around 0.3, which indicates the formation of complexes with a monodisperse distribution of particle size for both CS types and LNAs. Figure 5 shows the observed ζ-potentials in relation to N/P ratio which varies from −20 to +20 mV. All complexes with an N/P ratio of 10 showed a ζ-potential of +20 mV and further addition of CS did not induce a significant increase in the zeta potential.

### 2.2. Morphology of NPs

**Figure 6:**
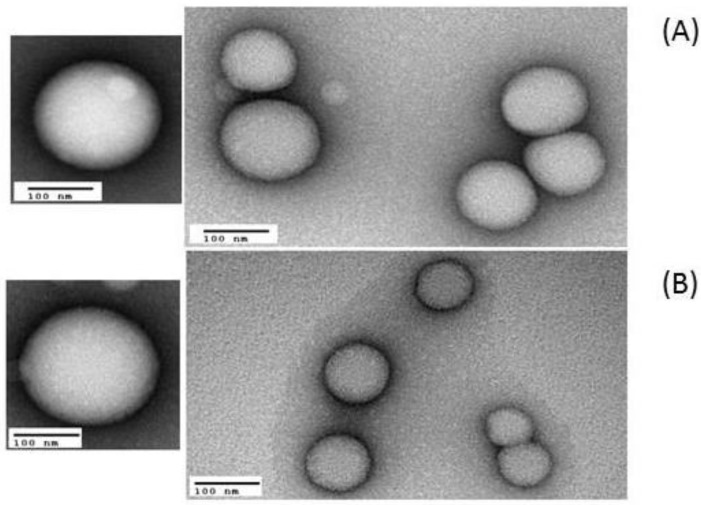
Representative TEM images of PLGA NPs containing (**A**) LNA1 and (**B**) LNA2 stained with UAR (Uranyl Acetate Replacement Stain).

**Figure 7:**
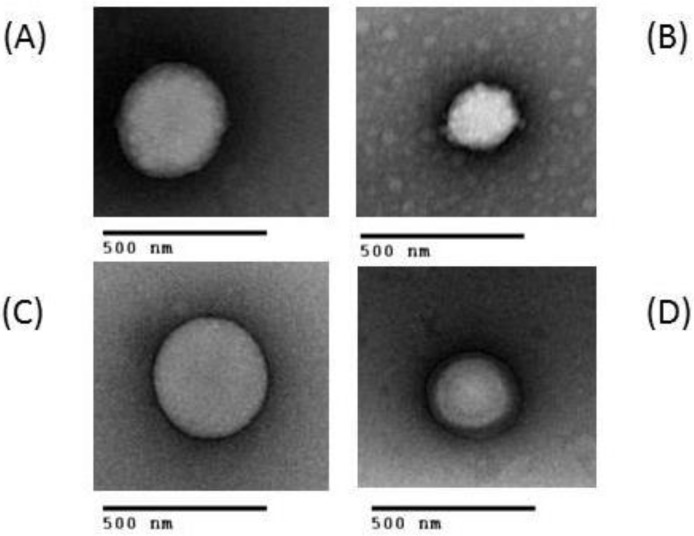
Representative TEM images of CS NPs at N/P ratio of 10 containing: (**A**) LNA1 and (**B**) LNA2 formed with CS Animal; and (**C**) LNA1 and (**D**) LNA2 formed with CS non-animal. All CS NPS were stained with UAR (Uranyl Acetate Replacement Stain).

Images of PLGA or CS NPs containing LNA1 and LNA2 were recorded by Transmission Electronic Microscopy (TEM) and analyzed in terms of particle morphology, size, and surface topology. Figure 6 shows representative images of PLGA containing LNA1 and LNA2. These values of size correlate well with those determined by Dynamic Light Scattering (DLS) and Nanoparticle Tracking Analysis (NTA). Figure 7 shows particles formed with all types of chitosans and LNAs at a ratio of 10. All nanoparticles were spherical in shape showing its heterogeneous nature. 

### 2.3. Encapsulation Efficiency

To accurately assess nucleic acid dosing and concentrations, the amount of each LNA encapsulated in the nanoparticles was determined by an RNA quantitation kit. This assay detects RNA in complex samples containing salts, free nucleotides, solvents, detergents, and protein. In our study, we have found that PLGA nanoparticle encapsulation efficiencies of LNA1 and LNA2 on average were found to be 70% and 20%, respectively.

### 2.4. Stability of CS-NPs in Biological Media

**Figure 8:**
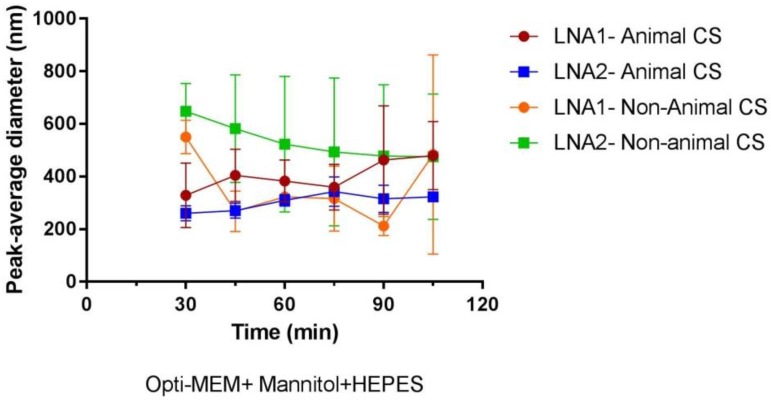
Stability of the LNA-CS NPs after initial incubation for 30 min and after every 15 min over the course of 105 min in Opti-MEM containing HEPES (4-(2-hydroxyethyl)-1-piperazineethanesulfonic acid) and mannitol at 37 °C. The values represented are the mean ± SD of three independent experiments.

The stability of different NP complexes during incubation in Opti-MEM containing HEPES and mannitol (equivalent to hypertonic conditions of 580 mM) was studied by DLS. Evolution of particle size distribution curves was analyzed after initial incubation at 37 °C for 30 min and after every 15 min during 105 min. Opti-MEM supplemented with HEPES and mannitol is recommended as a suitable transfection medium for Novafect (a CS-based commercial transfecting agent) from Novamatrix (Sandvika, Norway). At the same time, it is also well known that Opti-MEM is a commonly used medium to transfect epithelial cells [29]. Figure 8 shows the evolution of size over time for CS-LNA1 and CS-LNA2 from the two different CS sources. In general, an increase in average size diameter is observed when the particles are suspended in transfection medium. For particles formed with animal chitosan the diameter size increases over time from ~300 to ~400 nm. The opposite effect is observed for particles formed with non-animal chitosan, which have an initial particle size of ~600 nm and over time they tend to stabilize and the size decreases to values of ~450 nm.

### 2.5. NPs Nebulization

**Figure 9:**
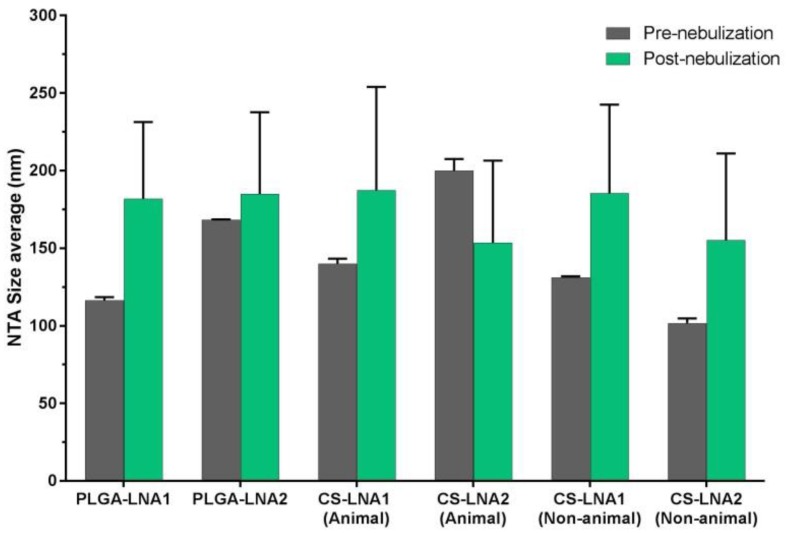
Effect of nebulization in LNAs-NPs. All LNA-NPs were measured before and after nebulization using NTA technique. The Aerogen Solo vibrating mesh nebulizer (Aerogen, Galway, Ireland) was used to generate the aerosol.

To verify suitability for aerosolization the different biopolymers used in this study, PLGA, animal chitosan and non-animal chitosan NPs loaded with LNAs were aerosolized using the vibratingmesh type nebulizer, the Aerogen Solo (Aerogen, Galway, Ireland). No changes in size were detected in any case confirming that the nanoparticles were intact and not adversely affected by the process of nebulization using a vibrating mesh nebulizer. To test the quality of NPs post-nebulization, the particle size of all NPs pre- and post-nebulization were determined (Figure 9). 

## 3. Discussion

Successful gene delivery is among the main challenges in lung CF gene therapy. This is attributed to the negative charge and hydrophilic character of nucleic acids. These characteristics prevent them from entering cells by passive diffusion. Classically, gene therapy vectors are broadly classified as viral or non-viral, but the latest ones are less immunogenic and easier to manufacture [30]. To find a safe and efficient non-viral vector for CFTR-specific Locked Nucleic Acids (LNAs) delivery, in this study, we described here the use of biocompatible and biodegradable biopolymer-based nanoparticles (NPs). We demonstrate the successful formulation and physicochemical characterization of LNAs nanoparticles based on either synthetic or natural cationic polymers, such as PLGA and chitosan (CS) from animal or non-animal source. The potential for aerosol-mediated delivery was also assessed.

Nanoparticle size and surface properties are key parameters influencing a delivery system in terms of safety, release kinetics, as well as tissue penetration and cellular internalization [31,32]. It is well reported that efficient intracellular internalization via endocytosis requires positively charged particles of less than 500 nm [33]. Larger particles are reported to be phagocytosed by macrophages [34]. Our study shows that LNAs formed particles are in the range of 200 nm as measured by using the common and widespread method of Dynamic Light Scattering (DLS) but also using a cutting edge technique such as Nanoparticle Tracking Analysis (NTA). A comparison of the data acquired using the two techniques is shown in Table 2. LNA-PLGA NPs showed a slightly smaller size (<200 nm) when NTA is used in comparison with DLS (~200 nm). In the case of LNAs-CS NPs, the average size observed for these systems is lower than 200 nm in all cases. It is well reported that NTA presents several advantages and benefits over DLS including the analysis of polydisperse systems and the concentration of nanoparticles. In addition, DLS measures changes in scattering intensity from a bulk sample, while NTA measures observed particle diffusion directly from particle-by-particle analysis [35]. NTA also has a substantially better peak resolution than DLS when the two methods are compared [36]. Likewise, images captured by TEM are in agreement with the results obtained by DLS and NTA. These images have allowed us to visualize the complexes and evaluate their morphology and spherical character.

Complexes formed with PLGA are all negatively charged, due to the presence of phosphate groups from the nucleic acids. Nonetheless, anionic systems have also been reported to produce successful therapeutic delivery [37]. Comparable evidence has already been reported in similar nanoparticles formed between PLGA and microRNA, where complexes presented a charge of −21.4 mV [38]. For particles formed with chitosans it is possible to observe that most of the complexes are negatively charged at low N/P ratios. Values of zeta potential increase, almost in a monotonic manner, with increasing N/P ratios. It is worth mentioning here that negative zeta potential values were not expected since this was previously calculated in order to produce positive complexes. However, it might be possible to argue that, because zeta potential is only an indicator of stability against agglomeration for charged stabilized systems, from our data we can speculate that complexes formed at low N/P ratios (e.g., below 5) might present a different conformation but they are still stable [39]. The trend observed in zeta potential values, might explain the increase in size for particles formed between LNA2 and animal chitosan at N/P ratio of 5. Under these conditions, zeta potential values reveal that the complexes are close to neutrality, the effect of repulsive forces is almost negligible and nanoparticles could have got together.

The formulation for PLGA NPs resulted in a high loading capacity for LNAs achieving levels of 70% and 20% for LNA1 and LNA2, respectively. Protocol optimization regarding the formulation of these PLGA NPs using the cationic lipid DOTAP based on previous studies carried out in our laboratory [20] allowed us to achieve these aforementioned levels of encapsulation efficiency. Such values are comparable to those reported in the literature using PLGA and siRNA [40].

Stability in transfection medium is a fundamental parameter for judging the behavior and effectiveness of particles in cell environment and later on their successful translation into clinical trials. In fact, there is a lack of systematic stability studies or methodologies for assessing the evaluation of this important parameter in in vitro experiments. The colloidal stability of NP in the cell environment will determine their biodistribution, pharmacokinetics, and systemic toxicity [41]. Here, it can be observed that chitosan nanoparticles remain relatively stable throughout the duration of the study e.g., for 105 min. After this time, it is expected that particles would be attached to the cell membrane by electrostatic forces and afterwards starts the internalization process via endocytosis. The observed increase in particle size in transfection medium is related to interactions with the proteins and electrolytes present in such environment. The stabilization over at least 105 min is due to the presence of repulsive hydration forces, these have been previously reported by the authors for chitosan particles [15]. However, changes among our different samples can be due to many factors like the thickness of the absorbed protein layer formed onto the particles and the ionic strength of the medium. A more detailed study would be required in order to recognize the potential influence of these parameters.

Aerosolized therapeutic treatments represent the ideal route of administration for a variety of lung diseases such as asthma, chronic obstructive pulmonary disease, respiratory infection and lung cancer [42]. In fact, nanoparticle suspensions can be delivered to the lungs via inhalation using nebulizers [43]. However, recent data shows that nanoparticles can be also delivered as dry powder formulations [44,45,46]. In the specific context of cystic fibrosis gene therapy studies, it has been previously documented that NPs can deliver genes to the airway epithelium by nebulization [47,48]. Here LNA-NPs formulated in water and nebulized by an Aerogen Solo vibrating mesh type nebulizer, which facilitates high efficiency dose delivery of aerosol medications to the lung [49,50], retained their biophysical properties in terms of particle size suggesting that aerosolized LNA-NPs may be suitable for cellular uptake and potentially useful for clinical application. 

Regarding the polymers used, nanoparticles formulated from PLGA offer a low toxicity and it is reported as an efficient carrier system for the delivery of nucleic acids in cells [51]. Based on previous literature, PLGA particles enter the cell efficiently through specific and non-specific endocytosis mechanism and PLGA nanoparticles can slowly release the encapsulated cargo leading to therapeutic effect [52]. In addition, PLGA particles have previously been tested for the delivery to the lungs, including pharmaceuticals currently used for the treatment of inflammatory lung diseases such as CF and COPD [53].

In the case of the natural polymer, chitosan is particularly interesting as a gene carrier due to its biodegradability, biocompatibility, low toxicity and low immunogenicity [6]. It is well documented that chitosan is a biotechnologically suitable transfecting system for gene therapy purposes [54]. Our previous studies provided a proof-of-principle that chitosan from animal sources was a natural non-toxic vector able to co-transfect a well-established model cell line for CF [15]. To the best of our knowledge this is the first time the use of chitosan from a non-animal source as a potential therapeutic vector has been reported. Here, LNA-NPs from a non-animal chitosan source, specifically from *Aspergillus niger* cell wall constituents, were fully characterized at the physicochemical level demonstrating it to be a potential new resource and highlighting it as an alternative to chitosan derived from shellfish products.

## 4. Materials and Methods

### 4.1. Preparation of CFTR-Specific LNAs-Loaded PLGA/DOTAP Nanoparticles

LNA oligonucleotides (Exiqon) were encapsulated in DOTAP/PLGA nanoparticles using the double emulsion solvent evaporation (DESE) method as previously described [40,55]. To improve encapsulation efficiency LNA was condensed with a cationic lipid DOTAP at an N/P (defined as the molar ratio of amine to phosphate groups) ratio of 4:1 using a hydration of freeze-dried matrix (HFDM). Briefly, CFTR-specific LNAs were diluted in 200 µL of RNA-free water and DOTAP was dissolved in 200 µL of Tertbutanol. The LNA solution was added dropwise to the lipid mixture, mixed, and lyophilized overnight. PLGA 503H (Boehringer Ingelheim, Ingelheim am Rhein, Germany) was dissolved in dichloromethane (DCM) (2.9% *w*/*v*) and vortexed. Lyophilized LNA/DOTAP was resuspended in RNase-free water and PLGA solution and sonicated for a total of 3 bursts of 5 s in continuous pulses mode at 70% amplitude to form the primary (water-in-oil (*w*/*o*)) emulsion. The primary emulsion was added dropwise to a 2% (*w*/*v*) poly(vinyl alcohol) (PVA) solution homogenizing on ice for 10 min in continuous pulses mode at 70% amplitude to form a secondary water-in-oil-in-water (*w*/*o*/*w*) emulsion and then added to 2% (*w*/*v*) PVA. The emulsion was mechanically stirred in the fume hood over night to allow the solvent to evaporate and allow nanoparticle formation. The nanoparticles were collected by centrifugation at 30,000 *g* for 25 min at 4 °C. To remove residual PVA, nanoparticles were washed in distilled water and centrifuged thrice. Following this, samples were resuspended in RNase-free water. Nanoparticles (1 mg) were freeze-dried for 24 h in 1 mL RNAse-free water.

### 4.2. Preparation of CFTR-Specific LNAs—Chitosan Nanoparticles

Ultra-pure biomedical grade chitosans were used to prepare the nanoparticles. Non-animal chitosan from *Aspergillus niger* was used for the formulation and it was provided by ChiPro GmbH (Bremen, Germany) (Batch No. 0151222) with a DA = 20%, Mw = 200 kDa based on the manufacturer’s specifications. Likewise, chitosan from an animal origin was provided by HMC+ (Halle, Germany; Code 70/5 Product No. 24200, Batch No. 212-170614-01; DA = 30%, Mw = 20 kDa based on the manufacturer’s specifications). 

The chitosans were dissolved in 1% acetic acid solution overnight at room temperature to a stock concentration of 2 mg/mL, and then diluted with ultra-pure water to reach the desired concentration. A series of complexes were prepared at different charge (N/P) ratios, (defined as the molar ratio of amine to phosphate groups) by mixing the chitosan working solutions with a constant amount of LNA (Table 1). The mixtures were incubated for 30 min at room temperature to form the self-assembled complexes.

### 4.3. Nanoparticle Characterization

The size distribution of the NPs was determined by dynamic light scattering with non-invasive back scattering (DLS-NIBS) at an angle of 173 with an automatic attenuator setting. The zeta potential (Z) was determined from the electrophoretic mobility by mixed-laser Doppler electrophoresis and phase analysis light scattering (M3-PALS), using the well-known Henry’s equation and Smoluchowski’s approximation as reported in previous studies [9,15,56]. Samples were prepared as previously described before being diluted to 1 ml. Both parameters were measured using a Malvern Zetasizer ZS 3000 (Malvern, UK). 

Nanoparticle tracking analysis (NTA) measurements were performed with a Nanosight NS300 (Malvern, UK). All suspensions were diluted in ultrapure water for measurement following equilibration. Samples were loaded into a laser module sample chamber which allowed for temperature control. Real time video analysis of the nanoparticles was recorded via an in-built sCMOS camera with computer controlled motorized focus. Automatic data analysis was performed on recorded data using the NTA 2.3 software. 

### 4.4. Encapsulation Efficiency of LNAs-PLGA NPs

Nanoparticle yield was measured by weighing a 1:10 aliquot lyophilised in a pre-weighed, non-static microtube on an MX5 microbalance (Mettler Toledo, Columbus, OH, USA). For measuring encapsulation efficiency (EC) and loading capacity (LC), a 1:10 aliquot (approx. 1 mg) of lyophilised NPs was dissolved in 200 µL chloroform for 10 min and subsequently vortexed at room temperature for 90 min after the addition of 500 µL dissociation buffer (1X Tris-EDTA, 2 mg/mL heparin and 0.292 mg/mL octyl ß-d-glucopyranoside). The vortexed emulsion was centrifuged at 26,000× *g* for 15 min at 4 °C. The aqueous layer was transferred to a new microtube and nucleic acid content was measured with the Quan-IT microRNA Assay kit (Thermo Fisher Scientific, Waltham, MA, USA). The measurements were controlled by performing parallel measurements with stock nucleic acid concentration dissolved in dissociation buffer.

(1) %EE=retrievednucleicacidloadednucleicacid×100

(2) LC(μgnucleicacid/mgNPs)=retrievednucleicacid(μg)NPs(mg)

### 4.5. NPs Morphology

Nanoparticles were visualized by transmission electron microscopy (TEM) in order to further confirm size and determined the morphology. Briefly, LNAs-NPs were prepared as previously described. A drop of NPs suspension was placed on a silicon monoxide carbon-coated copper grid (200 mesh, Mason technologies, Deer Park, NY, USA). Samples were allowed to air dry for approximately 10–15 min before being negative stained with 2% uranyl acetate alternative (URA) solution. Excess stain was removed using filter paper and the grids allowed to air dry fully before analysis. Imaging was performed using a Hitachi H-7650 Transmission Electron Microscope (Hitachi High Technologies, Berkshire, UK) at 120 kV.

### 4.6. Stability in Biological Media of LNAs-CS NPs

The short-term stability of LNA-CS NPs was assessed by preparation of particles as above mentioned with 100 mL of Opti-MEM (Life Technologies, Hong Kong, China), supplemented with Mannitol and HEPES, and subsequently incubating them for 105 min at 37 °C under agitation. The stability was evaluated by measuring the evolution of the hydrodynamic size by DLS.

### 4.7. Nebulization

The LNAs-NPs were reconstituted in RNAse-free water at room temperature. Nebulization was conducted with the Aerogen Solo vibrating mesh nebulizer (Aerogen, Galway, Ireland). The NPs dispersions were nebulized into a 15 mL centrifuge tube affixed to the nebulizer and collected after nebulization was complete. The average particle size before and after nebulization was measured as described above using the NTA system.

### 4.8. Statistical Analysis

Results are expressed as means ± SD. Statistical analysis was carried out using GraphPad Software Prism v7 (San Diego, CA, USA). Experiments were statistically analyzed using non-parametric tests using the Kruskal-Wallis test and Holm Sidak test. Differences were considered statistically significant when *p*-values (*) < 0.05. All experiments were conducted at least in triplicate and with at least three technical replicates per experiment.

## 5. Conclusions

Here we reported a comprehensive investigation of polymeric nanoparticles (NPs) as a novel method for the delivery of CFTR-specific LNAs. To this end, we described the physicochemical characterization of LNA-NPs using different biocompatible and biodegradable polymers; synthetic poly lactide-co-glycolide (PLGA) and natural chitosan (CS). PLGA-LNA NPs were produced at (+/−) charge ratio of 4, while electrostatic self-assembled CS-LNA NPs from two sources (animal and non-animal) were complexed in a range of (+/−) charge ratios (from 1.5 to 15). Size distribution, polydispersity index and zeta potential were determined by dynamic light scattering (DLS) and Nanoparticle Tracking Analysis (NTA). The average size diameter for PLGA-LNA NPs was ~200 nm, while CS-LNA NPs were <200 nm regardless of CS source. Transmission electronic microscopy images confirmed previous findings. All NPs had a monodisperse particle size distribution. The surface charge was determined from the electrophoretic mobility which revealed a neutral to slightly negative surface charge in PLGA-LNA NPs. At higher (+/−) charge ratios (10, 15), LNA-CS NPs reached a stable value of approximately +20 mV. Encapsulation efficiency analysis revealed a high amount of LNA encapsulated by PLGA NPs. The LNA-CS NPs size slightly increased in stability studies performed using biological media. All LNA-NPs were nebulized, and no differences were found in terms of particle size with measurements prior nebulization. In conclusion, our work shows that CFTR-specific LNA biopolymer-based nanoparticles represent a promising system for further development of new lung-targeted CF therapeutic approaches.

## Figures and Tables

**Table 1:** Physicochemical characteristics of the materials used for nanoparticle preparations.

Polymer	Molecular Weight (Da)	Degree of Acetylation (%)	Lactic Acid Units (%)	Origin
polylactide-co-glycolic acid	24,000–38,000	--	50	Synthetic
non-animal chitosan	200,000	20	--	*Aspergillus niger*
chitosan HMC+	20,000	30	--	Shrimp’s shell

**Table 2:** Comparison of the measurements performed by Dynamic Light Scattering (DLS) and Nanoparticle Tracking Analysis (NTA) regarding the average size diameter of the Locked-Nucleic Acid Nanoparticles (LNA-NPs).

Nanoparticles	DLS	NTA
PLGA + LNA1	260 ± 60 nm	166 ± 2 nm
PLGA + LNA2	222 ± 30 nm	168.3 ± 0.3 nm
Animal CS + LNA1	120 ± 30 nm	140 ± 3 nm
Non-animal CS + LNA1	150 ± 20 nm	131.1 ± 0.8 nm
Animal CS + LNA2	130 ± 20 nm	200 ± 8 nm
Non-animal CS + LNA2	170 ± 59 nm	102 ± 3 nm
